# Effects of In-Process Ultrasonic Vibration on Weld Formation and Grain Size of Wire and Arc Additive Manufactured Parts

**DOI:** 10.3390/ma15155168

**Published:** 2022-07-26

**Authors:** Jun Zhang, Yanfeng Xing, Jijun Zhang, Juyong Cao, Fuyong Yang, Xiaobing Zhang

**Affiliations:** School of Mechanical and Automobile Engineering, Shanghai University of Engineering Science, Shanghai 201620, China; zj17355135811@163.com (J.Z.); 13761116082@163.com (J.Z.); caojuyong86823@163.com (J.C.); yangfy09@163.com (F.Y.); xiaobingzh@hotmail.com (X.Z.)

**Keywords:** wire and arc additive manufacturing, ultrasonic vibration, amplitude, cavitation, grain size

## Abstract

Wire and arc additive manufacturing (WAAM) is a competitive technique, which enables the fabrication of medium and large metallic components. However, due to the presence of coarse columnar grains in the additively manufactured parts, the resultant mechanical properties will be reduced, which limits the application of WAAM processes in the engineering fields. Grain refinement and improved mechanical properties can be achieved by introducing ultrasonic vibration. Herein, we applied ultrasonic vibration to the WAAM process and investigated the effects of wire feed speed, welding speed, and ultrasonic amplitude on the weld formation and grain size during ultrasonic vibration. Finally, a regression model between the average grain size and wire feed speed, welding speed, and ultrasonic amplitude was established. The results showed that due to the difference in heat input and cladding amount, wire feed speed, welding speed, and ultrasonic amplitude have a significant influence on the weld width and reinforcement. Excessive ultrasonic amplitude could cause the weld to crack during spreading. The average grain size increased with increasing wire feed speed and decreasing welding speed. With increasing ultrasonic amplitude, the average grain size exhibited a trend of decreasing first and then increasing. This would be helpful to manufacture parts of the required grain size in ultrasonic vibration-assisted WAAM fields.

## 1. Introduction

Wire and arc additive manufacturing (WAAM) is a layered manufacturing process using wire as a filler material and an electric arc as the heat source. Compared with other additive manufacturing processes, including laser and electron beam, WAAM seems to be suitable for fabricating medium and large parts due to its high deposition rate, excellent energy efficiency, open deposition environment, and low capital and feedstock costs [1,2]. In WAAM, the process of generating electric arc can be mainly divided into three categories, namely gas tungsten arc welding (GTAW), gas metal arc welding (GMAW), and plasma arc welding (PAW) [3]. Of the three welding processes, the GMAW-based cold metal transfer (CMT) seems to be the most suitable for WAAM applications [4]. Compared with GMAW, the CMT welding process produces less heat, resulting in less spatter and controlled metal deposition rates [5]. Elrefaey et al. [6] found that joints deposited by the CMT process have better mechanical characteristics than conventional welding processes and produced the 7075-T6 joints without spatter, cracks, and low porosity.

Although WAAM has many advantages, it also has certain defects, such as oxidation, delamination, high residual stress, deformation, cracking, porosity, and surface finish, etc. [5,7,8,9]. To improve forming quality and mechanical properties, a wide range of ancillary processes were proposed by researchers including heat treatment [10,11,12,13], inter-pass cold rolling [14,15,16,17], inter-pass cooling [18], molten pool oscillation [19], and ultrasonic vibration.

For heat treatment, an appropriate post-process heat treatment process can reduce residual stress and improve mechanical properties, but an improper post-process heat treatment process would be counterproductive. For inter-pass cold rolling, it is only suitable for some regular parts, such as thin-walled parts, owing to the restriction of the cold rolling devices.

Cheng et al. [20] applied ultrasonic impact treatment and shot peening to three base metal samples, respectively. The results showed that the peak compressive residual stress exceeded the near-surface base metal material yield stress. Yuan et al. [21] obtained refined grains by ultrasonically stirring the molten pool with a probe located behind the arc. Tian et al. [22] found the introduction of synchronous ultrasonic vibration results in increased weld penetration and reinforcement and decreased contact angle. Wang et al. [23] found that as the ultrasonic frequency increases, the grain size becomes smaller, but results in an increase in porosity. Chen et al. [24] pointed out that dendrite fragmentation and heterogeneous nucleation caused by cavitation and acoustic streaming lead to grain refinement. Gorunov et al. [25] investigated the effect of frequency and power of ultrasonic-assisted direct energy deposition on the microstructure of the deposited Ti-6Al-4V parts. They found that ultrasonic vibration causes the transition from columnar grains to equiaxed grains and the location of the equiaxed grain regions can be changed by changing the frequency and power. Zhou et al. [26,27] developed a calculation model to predict the impact stress field and depth of plastic deformation zone under ultrasonic impact treatment and analyzed the stress and strain fields of ultrasonically impacted components by experimental and numerical methods. Chen et al. [28] concluded that ultrasonic vibration refines grains of the Cu-8Al-2Ni-2Fe-2Mn alloy. They found that parts with ultrasonic vibration obtain smaller grain sizes and better material dispersion than parts without ultrasonic vibration. Huu Phan et al. [29] pointed out that vibration frequency is the dominant factor in the ultrasonic vibration-assisted EDM process, which can achieve a higher material removal rate with better surface quality. Based on multi-objective optimization analysis, Huu Phan et al. [30] concluded that the workpiece-related low-frequency vibration in EDM leads to a significant improvement in surface quality. Ma et al. [31] presented that reduced penetration and increased contact angle of a single bead induced by ultrasonic vibration. The experimental results showed that the directional growth of coarse columnar grains of Inconel 625 parts is inhibited and grains are refined under ultrasonic vibration. Yuan et al. [32] realized the simultaneous coupling of ultrasonic vibration and the laser and wire additive manufacturing process. The ultrasonic frequency and amplitude were fixed at 20 kHz and 16 μm, respectively. The epitaxial growth trend of the prior-*β* crystal is blocked and refines the microstructure of the deposited Ti-6Al-4V alloy parts under the influence of ultrasonic vibration. Then, they [33] applied the simultaneous ultrasonic-assisted laser and wire additive manufacturing process to austenitic stainless steel, which also resulted in grain refinement. Todaro et al. [34] pointed out that the introduction of ultrasonic vibration increases many initial crystallites and reduces the temperature gradient in the bulk of the molten pool, leading to the formation of fine grains. Sun et al. [35] applied ultrasonic impact to the surface of deposited weld in time after deposition. The results showed that inter-layer ultrasonic impact leads to a refined microstructure of low carbon steel parts, reduced local stress concentration, enhanced mechanical properties, and improved anisotropy. Chen et al. [36] found that increased molten pool temperature induced by ultrasonic cavitation and convection results in a reduced temperature gradient, which increases the nucleation rate and supercooling in favor of fine grain formation.

Based on the abovementioned literature on ultrasonic-assisted additive manufacturing, it can be concluded that the introduction of ultrasonic vibration interrupts the growth of columnar crystals and promotes the transformation of coarse columnar grains to fine columnar grains or equiaxed grains, resulting in better mechanical properties. The ultrasonic vibration process is generally divided into three types. The first is the ultrasonic vibration acting on the weld that is synchronously coupled with the additive manufacturing process. The second is the fixed ultrasonic vibration acting on the substrate. The third is the ultrasonic vibration after welding, which is called ultrasonic impact treatment. The scope of application of the first ultrasonic vibration process is limited by synchronously coupled ultrasonic vibration equipment, so the structures of deposited components are generally simple and regular. The third ultrasonic vibration process is ultrasonic impact treatment after welding, so its influence on the weld microstructure is not as strong as the first two ultrasonic vibration processes. Therefore, this paper adopted the second ultrasonic vibration process.

At present, the research on ultrasonic-assisted additive manufacturing generally investigates the effect of ultrasonic vibration on the microstructure and mechanical properties of additively manufactured parts under the condition of fixed ultrasonic frequency and amplitude, and there are a few studies on the effect of ultrasonic frequency. Therefore, this paper adopted the second ultrasonic vibration process to study the effect of ultrasonic amplitude, wire feed speed, and welding speed on the weld morphology and microstructure under in-process ultrasonic vibration. The regression model of average grain size versus wire feed speed, welding speed, and ultrasonic amplitude was established. The effects of wire feed speed, welding speed, and ultrasonic amplitude on average grain size were analyzed.

## 2. Materials and Methods

### 2.1. Experimental Set-Up and Materials

**Figure 1:**
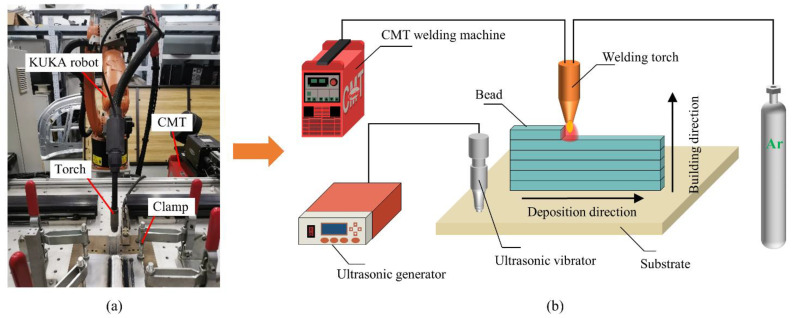
(**a**) Experiment platform of the wire and arc additive manufacturing (WAAM); (**b**) schematic diagram of WAAM assisted with ultrasonic vibration.

In the experiment, the WAAM system assisted with ultrasonic vibration consisted of three parts, namely the robot control system, the welding system, and the ultrasonic vibration system as shown in Figure 1. The KUKA KR5 6-axis robot (Augsburg, Germany) was employed to control the movement of the welding torch. The heat source of the welding system was supplied by the TPS4000-CMT welding machine (Fronius, Wels, Austria). A communication connection was established between the 6-axis robot and the welding machine. Thus, the start and end of the welding and the feeding of the welding wire can be controlled by the robot control system.

The welding wire used for deposition was ER4043 Al alloy wire with a diameter of 1.2 mm. The substrate material was 6061 Al alloy with dimensions of 200 mm × 180 mm × 5 mm. The chemical compositions of the welding wire and substrate material are shown in Table 1. Before WAAM, the substrate was wiped with alcohol to clean its surface stains. The surface was then sanded with sandpaper to remove the surface oxide film. The shielding gas (high-purity argon) flow rate was set at 20 L/min.

The ultrasonic vibration system consists of an ultrasonic generator, an ultrasonic vibrator, and a clamp. The ultrasonic vibrator was perpendicular to the contact surface of the substrate through the clamp, which ensured the effective supply of ultrasonic vibration to the substrate. The ultrasonic generator can convert alternating current into a high-frequency alternating current signal. Ultrasonic waves are formed when the output frequency exceeds 20 kHz.

**Figure 2:**
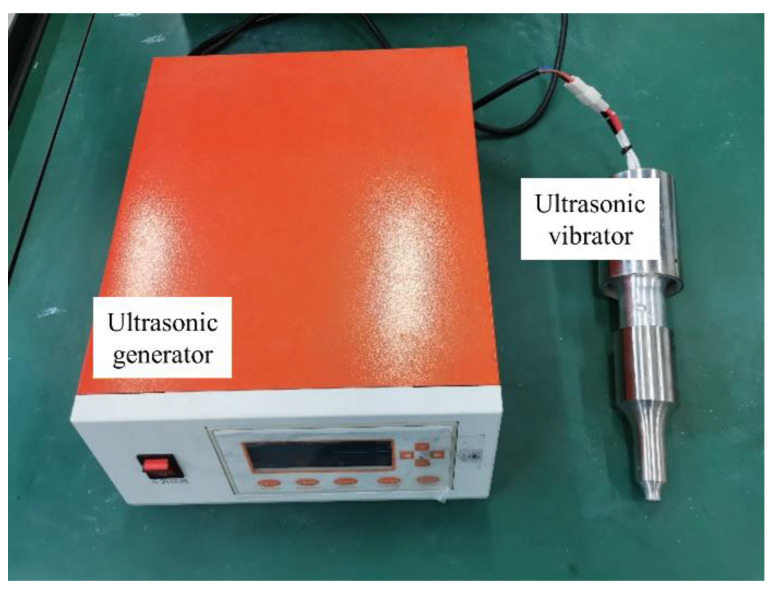
Ultrasonic generator and ultrasonic vibrator.

During the transmission of ultrasonic waves, there will be an alternating cycle of positive and negative pressures. When the ultrasonic waves are in a positive phase, the ultrasonic waves will squeeze the molecules of the medium, compressing the distance between the molecules to increase their density. When in a negative phase, the molecular spacing becomes larger and the density of the medium decreases. The ultrasonic vibrator is generally composed of ultrasonic transducers, horns, and tool heads. The ultrasonic transducer can convert the high-frequency electrical signal generated by the ultrasonic generator into mechanical power and transmit it with less mechanical loss. The horn increases the amplitude or velocity of mechanical vibration and focuses the ultrasonic vibration energy onto a smaller area. The function of the tool head is to directly contact the surface of the substrate and transmit the mechanical vibration to the molten pool. Due to the large energy generated by ultrasonic vibration, stainless steel, copper, or titanium alloys are generally used as the materials of the tool head. The tool head used in this experiment is made of stainless steel, and its shape is a circle with a diameter of 10 mm, as shown in Figure 2.

### 2.2. Experimental Method

In WAAM, the main welding parameters include wire feed speed, welding speed (torch travel speed), welding current, welding voltage, and shielding gas flow rate. Wire feed speed is directly related to welding current. The heat input increases with increasing wire feed speed, resulting in deeper penetration. Welding speed is related to the diffusion of heat. This will affect bead size and penetration. For ultrasonic vibration auxiliary equipment, the variable parameters mainly include frequency, amplitude, and distance between the ultrasonic vibrator and the beads, etc.

**Figure 3:**
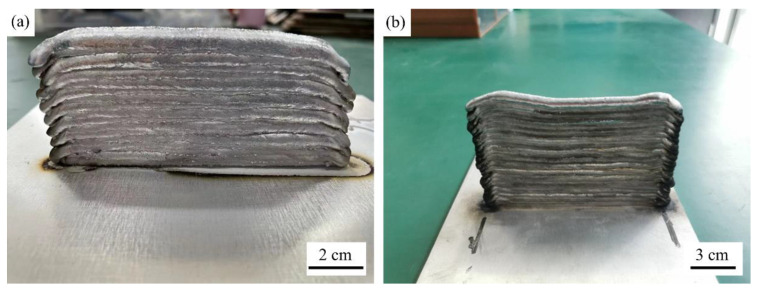
(**a**) Thin-walled parts fabricated with ultrasonic vibration; (**b**) thin-walled parts fabricated without ultrasonic vibration.

To study the effect of in-process ultrasonic vibration on wire and arc additive manufactured parts, the single-bead multi-layer thin-walled samples were fabricated. The welding torch followed a reciprocating deposition path to counteract the bulge at the start of the arc and sag at the end of the arc (shown in Figure 3). The stick out of the wire was 12 mm. The ultrasonic generator was turned on before welding and turned off after each layer deposition. The distance between the ultrasonic vibrator and the beads was set to 105 mm.

Since additive manufacturing is a layer-by-layer accumulation process; the study of the forming laws of single-bead single-layer parts is the basis for the deposition of the single-bead multi-layer parts. Therefore, in the paper, the one-factor-at-a-time method was adopted to study the effect of welding parameters on the formation of single-bead single-layer parts under ultrasonic vibration. Excluding the unstable arc-starting point and arc-extinguishing point, the weld width and reinforcement of the stable part of each weld were measured three times by the vernier caliper. The studied welding parameters include wire feed speed, welding speed, and ultrasonic amplitude. Based on the experimental results, the mechanism of ultrasonic vibration on the spreading motion of the weld pool was revealed.

Then, the single-bead multi-layer thin-walled parts were fabricated with and without ultrasonic vibration. To further observe the microstructure of the sample, the deposited thin-walled parts were cut with a wire electric discharge machine, then ground, polished, etched, and finally cleaned with alcohol to obtain metallographic samples. The microstructure was observed using a Leica CM4M optical microscope (Leica Microsystems, Wetzlar, Germany), and grain size measurements were performed using Image-Pro Plus software. The BBD (Box–Behnken) method was used to carry out the experimental design of the deposition of thin-walled parts and the regression model of average grain size versus wire feed speed, welding speed, and ultrasonic amplitude was obtained.

### 2.3. Determination of Ultrasonic Vibration Frequency

Since the ultrasonic amplitude is the main parameter studied in this paper, it is necessary to determine the appropriate ultrasonic frequency before the experiment. In the experiment, the ultrasonic vibrator applied mechanical vibration to the surface of the substrate, and the ultrasonic vibration was transmitted into the molten pool through the substrate, which stirred the molten pool.

Two nonlinear effects of cavitation and acoustic streaming are caused by the introduction of periodic positive and negative pressure and violent motion in the molten pool by ultrasonic vibration. The reduction of pores in the molten pool and the reduction of the average grain size are mainly attributed to ultrasonic cavitation. Cavitation results in the creation of tiny bubbles or small cavities, which then undergo a series of processes including inertial growth, pulsation, and collapse in the molten pool [37]. When the bubble collapses, high temperature and pressure will be generated inside the bubble in a very short time, and a strong shock wave will be generated instantly, causing the stirring and mixing of the molten pool. Cavitation occurs when the acoustic pressure exceeds the cavitation threshold.

The following Rayleigh–Plesset equation describes the nonlinear response of the bubble in a fluid to a driving pressure field [38].

(1) $$R\ddot{R}+\frac{3}{2}{\dot{R}}^{2}=\frac{1}{\rho }\left[P_{v}-P_{\infty }+P_{g0}{\left(\frac{R_{0}}{R}\right)}^{3k}-\frac{2\sigma }{R}-\frac{4\mu \dot{R}}{R}\right]$$

where *R* is the radius of the bubble at time *t*, *R*_0_ is the initial bubble radius, *ρ* is the liquid density, *σ* is the surface tension, *μ* is the dynamic viscosity of the liquid, *k* is the polytropic constant for gas, *P_v_* is the vapor pressure in the bubble, *P_∞_* is the pressure at infinity, and *P_g_*_0_ is the gas pressure at the reference radius *R*_0_.

In acoustic cavitation, owing to the effect of the acoustic field on the growth and collapse of bubbles, the *P_∞_* and *P_g_*_0_ should be modified [39,40].

(2) $$R\ddot{R}+\frac{3}{2}{\dot{R}}^{2}=\frac{1}{\rho }\left[P_{v}-\left(P_{0}+P\left(t\right)\right)+\left(P_{0}-P_{v}+\frac{2\sigma }{R}\right){\left(\frac{R_{0}}{R}\right)}^{3k}-\frac{2\sigma }{R}-\frac{4\mu \dot{R}}{R}\right]$$

where *P*_0_ is the static pressure and *P(t)* is the applied acoustic field.

Numerical simulation research based on Equation (2) found that lower ultrasonic frequency promotes ultrasonic cavitation. Tian et al. [41] found that with the increase of ultrasonic frequency, the period of cavitation bubbles becomes longer. Huang et al. [42] pointed out that with the increase of ultrasonic frequency, the amplitude of cavitation bubbles decreases, and the higher the frequency, the faster the attenuation of ultrasonic energy. Therefore, more energy is required to provide the same intensity of cavitation. Wei et al. [43] found that the radius of the cavitation bubbles increases to about 109, 80, 67, 32, 15, and 8 times the initial size during the ultrasonic frequency from 15, 20, 30, 50, and 100 to 200 kHz, respectively. The corresponding collapse period also increases. Experiments showed that the cavitation bubbles are transient and they shrink and collapse within 1.2 periods when the ultrasonic frequency is lower than 20 kHz, while the cavitation bubbles are stable and need more periods to shrink, expand, and collapse when the ultrasonic frequency is higher than 30 kHz. This is because for high-frequency ultrasonic vibration, the expansion time of the bubble is shortened, and the bubble radius does not have enough time to reach the cavitation threshold, resulting in too little time available for the bubble to collapse. It therefore periodically experiences collapse, expansion, oscillation, and collapse. Therefore, the ultrasonic vibration frequency was selected as 20 kHz in this paper.

## 3. Results and Discussion

### 3.1. Effect of Ultrasonic Amplitude on Weld

To study the effect of ultrasonic amplitude on the forming of the single-bead single-layer weld, the wire feed speed *V_w_* and welding speed *V_t_* were kept unchanged (*V_w_* = 4.5 m/min, *V_t_* = 0.3 m/min), and only the ultrasonic amplitude was changed. The specific welding parameter settings of the ultrasonic amplitude experiment are shown in Table 2.

**Figure 4:**
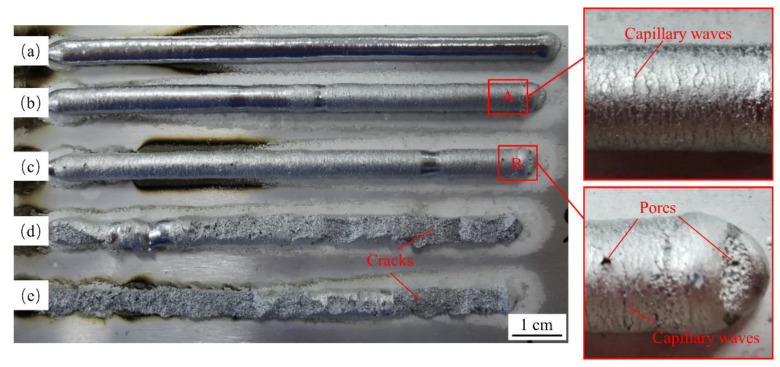
Effect of different ultrasonic amplitudes on weld: (**a**) sample 1; (**b**) sample 2; (**c**) sample 3; (**d**) sample 4; (**e**) sample 5.

As shown in Figure 4, the surface of sample 1 without ultrasonic vibration is covered with the metallic luster, its weld width is 5 mm, and reinforcement is 4.2 mm. Compared with sample 1, the surface of sample 2 obviously loses the metallic luster and capillary waves appear (shown in the enlarged view of area A). Its weld width is 5.7 mm and reinforcement is 4 mm. Capillary waves are generated by the violent oscillation of the molten pool caused by ultrasonic vibration. However, the capillary wave is small in size and will be remelted by the next deposition, so it does not affect the formed appearance [44]. A small number of tiny pores appear on the surface of sample 3, and a larger number of pores gather on the surface at the arc-end point. Its weld width is 5.8 mm and reinforcement is 4 mm. It can be seen in the enlarged view of area B that the capillary waves on the surface of sample 3 are more obvious than that of sample 2, which indicates that the ultrasonic vibration of sample 3 is more severe. The pores on the surface are generated because the bubbles inside the molten pool escape upward under the action of ultrasonic vibration, and the bubbles that do not fully escape before the solidification of the metal molten pool form open pores. It can be concluded from samples 1, 2, and 3 that the ultrasonic vibration makes the weld width wider and the reinforcement lower. The enlarged weld width is attributed to the stirring and oscillation effect of ultrasonic vibration on the molten pool, which accelerates the flow inside the molten pool. Due to the same wire feed speed and welding speed, the deposition amount of the wire per unit time is equal. Thus, the increase in the weld width will naturally reduce the reinforcement.

**Figure 5:**
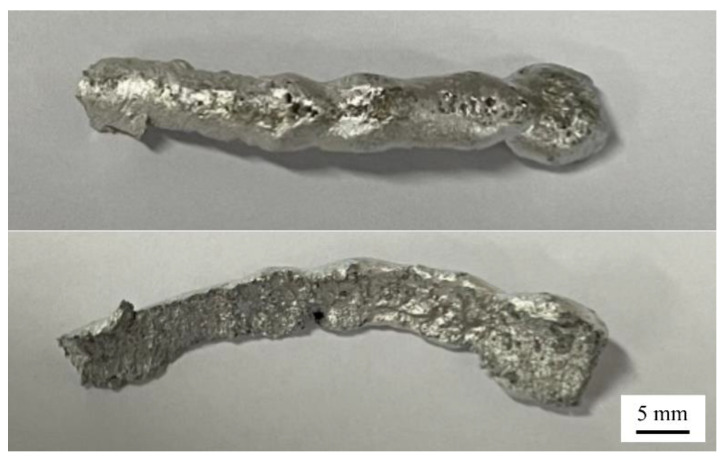
Part of the collapsed structure.

As shown in Figure 4d,e, it can be seen that the incompletely solidified weld cracks because of the excessive energy of ultrasonic vibration. Part of the cracked structure is shown in Figure 5. It can be seen that the cracked structure has begun to take the shape of the weld, which indicates that the crack occurs in the spreading stage of the molten pool. The greater the ultrasonic amplitude, the greater the energy generated by the ultrasonic vibration. Thus, when the molten pool is not completely solidified, the weld will crack when the tensile stress generated by ultrasonic vibration during the vibration period exceeds the ultimate tensile strength that can be endured in this state. Therefore, it is necessary to select the appropriate ultrasonic amplitude to achieve good weld formation when applying ultrasonic vibration.

### 3.2. Effect of Wire Feed Speed and Welding Speed on Weld

In the experiment, the wire feed speed was set to 4 m/min, 5 m/min, 6 m/min, and 7 m/min respectively, and the welding speed was set to 0.3 m/min, 0.45 m/min, and 0.6 m/min respectively. Based on the results of Section 3.1, the ultrasonic amplitude was chosen to be 20 μm. The specific welding parameter settings of the wire feed speed and welding speed experiment are shown in Table 3.

**Figure 6:**
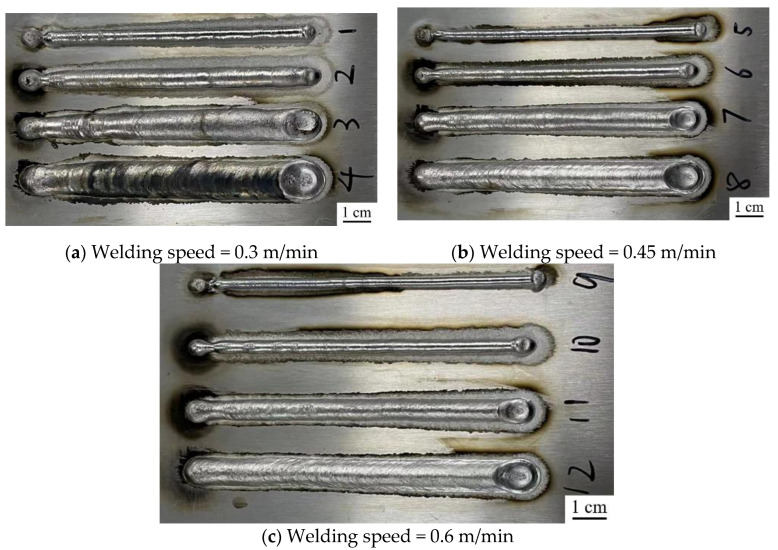
Weld macromorphology of the wire feed speed and welding speed experiment: (**a**) samples 1, 2, 3, and 4; (**b**) samples 5, 6, 7, and 8; (**c**) samples 9, 10, 11, and 12.

**Figure 7:**
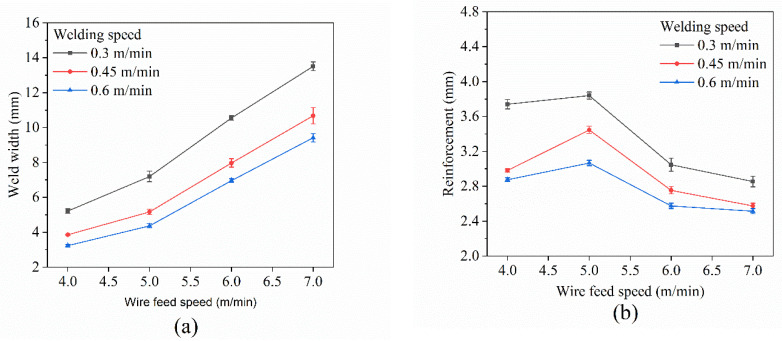
Effects of wire feed speed and welding speed on weld width (**a**) and reinforcement (**b**).

Figure 6 shows the weld macromorphology of the wire feed speed and welding speed experiment. Figure 7 displays the effects of wire feed speed and welding speed on weld width and reinforcement. As shown in Figure 6 and Figure 7, with the increase in wire feed speed, it can be clearly seen that the weld width increases, while the reinforcement first increases slightly and then decreases. The analysis was carried out with the experiment data at the welding speed of 0.45 m/min. When the wire feed speed (*V_w_*) was 4 m/min, the average weld width was only 3.85 mm, and the reinforcement was 2.98 mm. When *V_w_* = 5 m/min, the average weld width was 5.17 mm, and the reinforcement was 3.45 mm. When *V_w_* = 6 m/min, the average weld width increased to 7.97 mm, and the reinforcement decreased to 2.75 mm. The arc ending point of the weld was obviously depressed. When *V_w_* = 7 m/min, the weld width could reach 10.67 mm, and the reinforcement was only 2.57 mm. The obvious cracks appeared on the surface of the arc ending point depression. Two main parameters affect weld formation: one is the ratio of wire feed speed to welding speed, which represents the amount of welding wire deposited per unit time and unit length, and the other is heat input, which directly affects the melting of the welding wire and the substrate, and has a great influence on the flow of the molten pool. Therefore, we analyze and explain the weld formation from these two aspects.

The heat input can be calculated with the following equation:

(3) $$\mathrm{Heat}\;\mathrm{input}\;\left(J/\mathrm{mm}\right)=\frac{\eta UI}{V_{t}}$$

where *η* is the arc thermal efficiency, which was set to 0.9 in this experiment, *U* (V) is the welding voltage, *I* (A) is the welding current, and *V_t_* (m/min) is the welding speed. Wire feed speed is directly related to welding current. As the wire feed speed increases, the welding current and voltage increase, so the heat input increases. Higher heat input means longer molten pool solidification time and reduced molten pool viscosity, resulting in better molten pool fluidity. Meanwhile, the ultrasonic vibration induces agitation and oscillation of the molten pool, which contributes to the flow of the molten pool. Thus, the weld width increases. For the reinforcement, when the welding speed is the same; the increase in heat input from wire feed speed of 4 m/min to 5 m/min is small (shown in Table 3), but the increased amount of wire is sufficient. Therefore, when the wire feed speed increases from 4 m/min to 5 m/min, both the weld width and reinforcement are increased.

It is worth noting that the slopes are not the same in Figure 7, which reflects the degree of increase in heat input. A large slope means a large increase in heat input, which results in better molten pool flow. Thus, a wider weld width and a lower reinforcement are obtained.

As shown in Figure 7, it can be seen that with the increase in welding speed, both the weld width and reinforcement decrease when keeping the wire feed speed constant. For weld width, when the welding speed increases, and the amount of metal deposition per unit length decreases. Meanwhile, according to Equation (3), the heat input per unit length decreases, resulting in a weakened molten pool fluidity. Therefore, the weld width is reduced. For reinforcement, reduced metal deposition amount results in smaller reinforcement, while reduced molten pool fluidity results in larger reinforcement. However, it is clear that metal deposition amount is dominant among these two factors, so the reinforcement is reduced in the end.

### 3.3. Effect of Ultrasonic Vibration on the Average Grain Size of the Microstructure

To study the effect of ultrasonic vibration on the average grain size of the microstructure, the single-bead multi-layer thin-walled parts were fabricated (shown in Figure 3). When the total number of deposited layers is the same, due to the stirring and oscillation of the molten pool caused by ultrasonic vibration, the layer height of the thin-walled part with ultrasonic vibration is lower and its layer width is wider than that of the thin-walled part without ultrasonic vibration. The microstructure of thin-walled parts was analyzed, and the regression model of wire feed speed, welding speed, ultrasonic amplitude, and average grain size was established. A good prediction of the average grain size of WAAM of aluminum alloy parts was achieved.

Based on the results of Section 3.1, when the ultrasonic amplitude was 30 μm, the weld cracked under excessive ultrasonic energy. Therefore, the maximum ultrasonic amplitude in this experiment was set to 28 μm. The shielding gas flow rate was 20 L/min. Each sample was deposited with 6 layers, and the middle was taken to make a metallographic sample. The grain size measurements were taken using Image-Pro Plus software.

The BBD (Box–Behnken) method was applied to obtain the average grain size regression model. Each factor took three levels and encoded them with (−1, 0, +1), where 0 is the center point and –1 and +1 represent the upper and lower limits, respectively. The coding generated by Design Export software is given in Table 4 based on the single-bead single-layer experiment. The resulting experimental design matrix is shown in Table 5. After 17 metallographic samples were prepared, each sample was observed and the average grain size was measured. In order to ensure the validity of the test data, each sample was intercepted at two different positions for observation.

**Figure 8:**
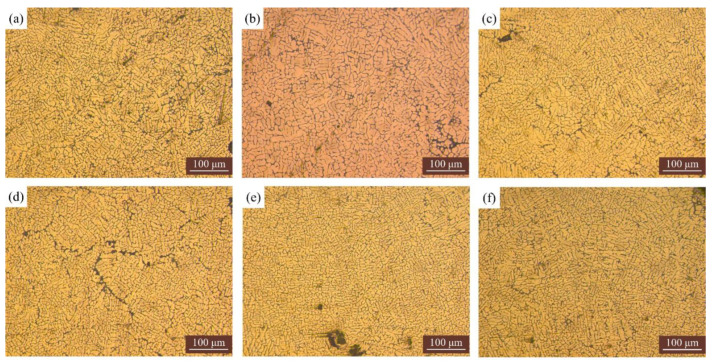
Microstructure of some samples: (**a**) Exp. 1; (**b**) Exp. 5; (**c**) Exp. 7; (**d**) Exp. 8; (**e**) Exp. 10; (**f**) Exp. 13.

The microstructure of some samples is shown in Figure 8. Based on our previous research, it can be concluded that there were a lot of columnar crystals in the microstructure of the sample without ultrasonic vibration. Due to the excessively large temperature gradient during the WAAM, a large number of dendrite structures existed, and a large amount of Al-Si eutectic structure existed in the interstitial space of the dendrite structure. The aluminum eutectic structure in the absence of ultrasonic vibration was mainly in the form of long strips. Our previous research showed that the average grain size of the sample without ultrasonic vibration was measured to be 30.2 μm, and the largest grain size could reach 101.98 μm. As shown in Figure 8, it can be seen that the microstructure of the samples with ultrasonic vibration was mainly equiaxed crystals, with a small number of columnar crystals existing. Their average grain size is smaller than that of the samples without ultrasonic vibration (shown in Table 5). It is worth noting that our previous studies have shown that excessive ultrasonic amplitude can lead to larger grain size and cracks. It may be because as the ultrasonic amplitude increases, the energy generated by the ultrasonic waves in the medium is higher, and the increase in heat input causes the cooling rate inside the molten pool to slow down, resulting in grain coarsening.

The regression equation was obtained as follows:

(4) $$\begin{array}{cc} Average\;grain\;size & \\ = & 126.275-17.2315A-168.5505B-3.2308C+3.5AB\\ - & 0.05625AC+1.51875BC+1.8194A^{2}+125.485B^{2}+0.062647C^{2} \end{array}$$

The ANOVA results of the regression model are shown in Table 6. The model *F*-value of 28.64 implies the model is significant. There is only a 0.01% chance that an *F*-value this large could occur due to noise. The *p*-values less than 0.05 indicate model terms are significant. In this case, *A*, *B*, *C*, *BC*, *A*^2^, *B*^2^, and *C*^2^ are significant model terms. Values greater than 0.1000 indicate the model terms are not significant. The Lack of fit *F*-value of 6.56 is not significant. The coefficient of determination *R^2^* of 0.9736 means that the regression model clarifies 97.36% of all deviations.

**Figure 9:**
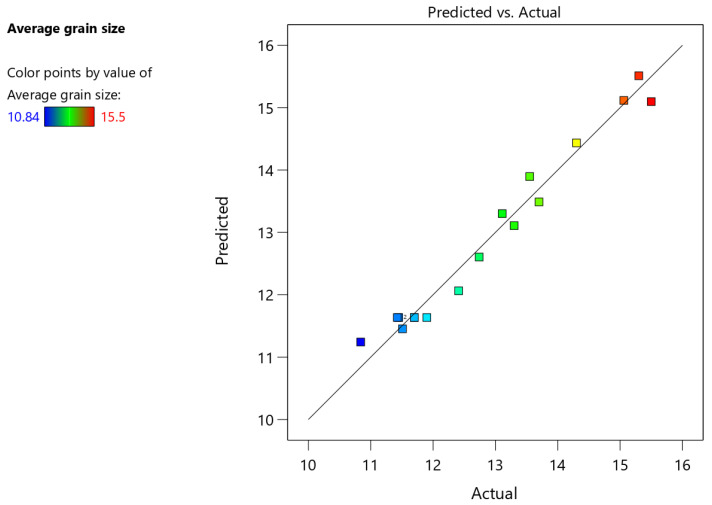
Comparison of predicted grain size with actual grain size.

Figure 9 shows a comparison of the average grain size predicted by Equation (4) with the actual measured value. When the predicted value is closer to the actual value, the points will be closer to the line. It can be seen from Figure 9 that the most of points are distributed near the straight line, indicating that Equation (4) can well reflect the influence of each welding parameter on the average grain size.

**Figure 10:**
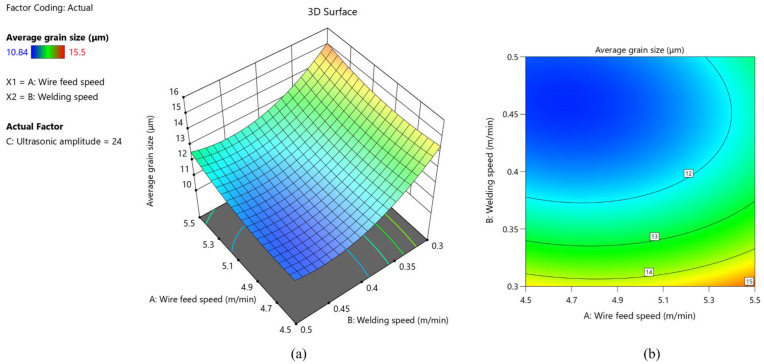
(**a**) 3D response surface of the interaction between wire feed speed and welding speed; (**b**) 2D contour of the interaction between wire feed speed and welding speed.

**Figure 11:**
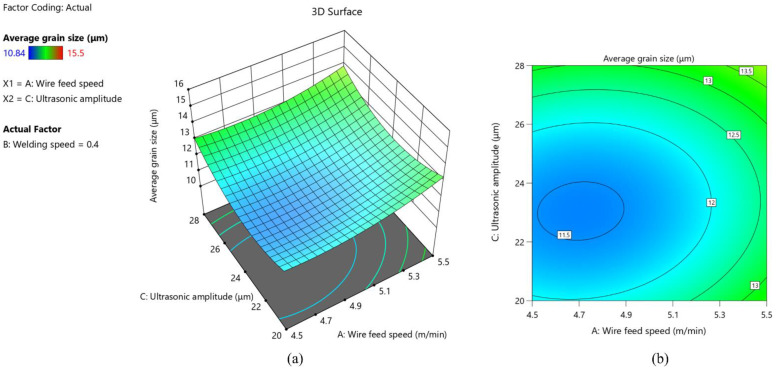
(**a**) 3D response surface of the interaction between wire feed speed and ultrasonic amplitude; (**b**) 2D contour of the interaction between wire feed speed and ultrasonic amplitude.

**Figure 12:**
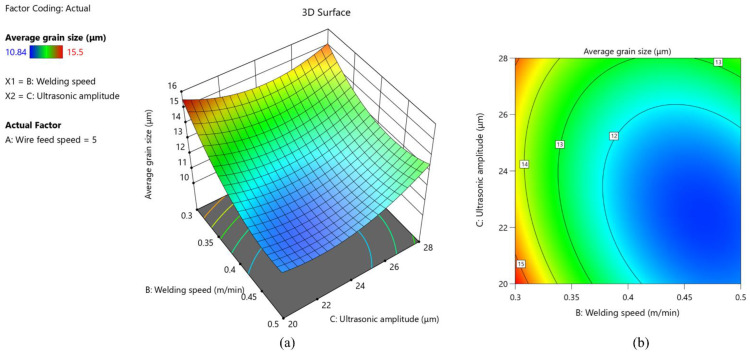
(**a**) 3D response surface of the interaction between welding speed and ultrasonic amplitude; (**b**) 2D contour of the interaction between welding speed and ultrasonic amplitude.

As shown in Figure 10, the average grain size keeps getting larger as wire feed speed increases and welding speed decreases. In the case of high wire feed speed and low welding speed, the amount of wire melted into the molten pool per unit length reaches the maximum, resulting in high heat input. At the same time, the low welding speed makes the welding heat source stay above the molten pool for too long, and the cooling speed is low. These two reasons lead to the growth of grains inside the weld under the influence of prolonged heat input. It can be clearly seen from Figure 11 that the interaction of ultrasonic amplitude and wire feed speed has no significant effect on the average grain size. As shown in Figure 12, the larger slope of the 3D response surface and the elliptical 2D contour indicates that the interaction of ultrasonic amplitude and welding speed on the average grain size is significant. It can be seen that with the increase of ultrasonic amplitude, the average grain size shows a trend of decreasing first and then increasing.

Based on the above analysis, the lack of fit *F*-value of 6.56 and *p*-value of 0.0504 are not very good. Therefore, we removed the insignificant terms to reduce the impact on the model, resulting in the following simplified regression model:

(5) $$\begin{array}{cc} Average\;grain\;size & \\ = & 126.025-17.1815A-151.0505B-3.51205C\\ + & 1.51875BC+1.8194A^{2}+125.485B^{2}+0.062647C^{2} \end{array}$$

## 4. Conclusions

In this study, the single-bead single-layer and single-bead multi-layer aluminum alloy components were fabricated by in-process ultrasonic vibration-assisted WAAM. The effects of wire feed speed, welding speed, and ultrasonic amplitude on the weld formation and microstructure grain size were investigated. The following conclusions can be drawn:

1. With the increase of ultrasonic amplitude, the weld width became wider and the reinforcement becomes lower. When the ultrasonic amplitude was too large and exceeded the ultimate tensile strength that could be endured in the state, the weld cracked without fully spreading.
2. Under ultrasonic vibration with an amplitude of 20 μm, increased welding speed resulted in reduced weld width and reinforcement. With the increase of wire feed speed, the weld width increased, and reinforcement first increased slightly and then decreased.
3. Average grain size decreased with decreasing wire feed speed and increasing welding speed. When the ultrasonic amplitude increased, the average grain size first decreased and then increased.
4. The significant effect of the interaction between parameters on the average grain size is as follows: welding speed and ultrasonic amplitude > wire feed speed and welding speed > wire feed speed and ultrasonic amplitude.

## Figures and Tables

**Table 1:** Chemical compositions of the wire and substrate (wt. %).

Material	Si	Fe	Cu	Mn	Mg	Zn	Cr	Ti	Al
ER4043	4.5–6	0.8	0.3	0.05	0.05	0.1		0.2	Bal.
6061 Al	0.4–0.8	0.7	0.15–0.4	0.15	0.8–1.2	0.25	0.04–0.35	0.15	Bal.

**Table 2:** Specific welding parameter settings of the ultrasonic amplitude experiment.

S.NO.	Wire Feed Speed (m/min)	Welding Speed (m/min)	Amplitude (μm)	Shielding Gas Flow Rate (L/min)
1	4.5	0.3	0	20
2	4.5	0.3	20	20
3	4.5	0.3	25	20
4	4.5	0.3	30	20
5	4.5	0.3	35	20

**Table 3:** Specific welding parameter settings of the wire feed speed and welding speed experiment.

S. NO.	Wire Feed Speed (m/min)	Welding Speed (m/min)	Welding Voltage (V)	Welding Current (A)	Heat Input (J/mm)	Amplitude (μm)	Shielding Gas Flow Rate (L/min)
1	4	0.3	12	69	149.04	20	20
2	5	0.3	12.6	85	192.78	20	20
3	6	0.3	15.3	127	349.758	20	20
4	7	0.3	16.8	158	477.792	20	20
5	4	0.45	12	69	99.36	20	20
6	5	0.45	12.6	85	128.52	20	20
7	6	0.45	15.3	127	233.172	20	20
8	7	0.45	16.8	158	318.528	20	20
9	4	0.6	12	69	74.52	20	20
10	5	0.6	12.6	85	96.39	20	20
11	6	0.6	15.3	127	174.879	20	20
12	7	0.6	16.8	158	238.896	20	20

**Table 4:** Coding for factor and level.

Factor Level	Wire Feed Speed (m/min)	Welding Speed (m/min)	Ultrasonic Amplitude (μm)
−1	4.5	0.3	20
0	5	0.4	24
+1	5.5	0.5	28

**Table 5:** Experiment design matrix and the response.

Exp. NO.	Wire Feed Speed (m/min)	Welding Speed (m/min)	Ultrasonic Amplitude (μm)	Average Grain Size (μm)
1	4.5	0.4	28	13.3
2	5	0.4	24	11.4265
3	4.5	0.3	24	14.3
4	5	0.3	20	15.3
5	5.5	0.3	24	15.5
6	4.5	0.4	20	12.41
7	5.5	0.4	20	13.11
8	5	0.4	24	11.7
9	5	0.5	28	13.7
10	4.5	0.5	24	10.84
11	5.5	0.5	24	12.74
12	5	0.4	24	11.9
13	5	0.4	24	11.45
14	5	0.3	28	15.06
15	5	0.4	24	11.7
16	5.5	0.4	28	13.55
17	5	0.5	20	11.51

**Table 6:** ANOVA results of the regression model.

Source	Sum of Squares	Mean Square	*F*-Value	*p*-Value
Model	34.08	3.79	28.64	0.0001
*A*-wire feed speed	2.05	2.05	15.51	0.0056
*B*-welding speed	16.16	16.16	122.24	<0.0001
*C*-ultrasonic amplitude	1.34	1.34	10.17	0.0153
*AB*	0.1225	0.1225	0.9267	0.3678
*AC*	0.0506	0.0506	0.3830	0.5556
*BC*	1.48	1.48	11.17	0.0124
*A* ^2^	0.8711	0.8711	6.59	0.0372
*B* ^2^	6.63	6.63	50.15	0.0002
*C* ^2^	4.23	4.23	32.00	0.0008
Residual	0.9253	0.1322		
Lack of fit	0.77	0.26	6.56	0.0504

## Data Availability

The data used to support the findings of this study can be made available upon reasonable request from the corresponding author.
